# Telesimulation for Training in Infant Feeding: A Randomized Controlled Trial

**DOI:** 10.1007/s00455-024-10746-7

**Published:** 2024-08-12

**Authors:** Jeanne Marshall, Charis Shiu, Madeline Raatz, Adriana Penman, Kelly Beak, Sally Clarke, Elizabeth C. Ward

**Affiliations:** 1 Queensland Children’s Hospital, Children’s Health Queensland Hospital and Health Service, Brisbane, Australia; 2 School of Health and Rehabilitation Sciences, The University of Queensland, Brisbane, Australia; 3 Logan Hospital, Metro South Hospital and Health Service, Brisbane, Australia; 4 Centre for Functioning and Health Research (CFAHR), Metro South Hospital and Health Service, Brisbane, Australia

**Keywords:** Bottle feeding, Patient simulation, Pediatric feeding disorder, Speech–language pathology, Telepractice, Training

## Abstract

Simulation is an education modality known to support clinical skill development. Unfortunately, access to simulation has been challenging, both prior to and during the pandemic. Simulation via telepractice, i.e., “telesimulation”, has emerged, but little is known about whether outcomes are comparable to in-person simulation. This study compared in-person versus telesimulation learner outcomes in an infant feeding scenario. The secondary aim was to compare outcomes between novice and experienced participants.

This pragmatic randomized controlled trial included speech pathologists who could attend if randomized to the in-person modality. Block randomization matched participants with < 6 months’ infant feeding experience to those with > 6 months experience (2:1 ratio) into telesimulation or in-person simulation. Measures of clinical reasoning, confidence/anxiety, and satisfaction were collected, pre-, post-, and 4-weeks post-simulation.

Overall, 39 clinicians completed either in-person simulation (*n* = 17) or telesimulation training (*n* = 22), including 16 experienced and 23 novice learners. Both in-person and telesimulation groups achieved significant improvements across time in clinical reasoning, self-reported confidence, and anxiety. The extent of change in clinical reasoning, confidence and anxiety was comparable between the telesimulation and in-person simulation groups. Comparing by experience, novice-level participants reported significantly greater changes in confidence and anxiety than experienced participants. Satisfaction levels were high regardless of simulation modality or experience.

Participants in telesimulation and in-person simulation achieved similar improvements in the primary outcome measure of clinical reasoning, had comparable improvements in self-perceived confidence and anxiety, and demonstrated high satisfaction levels. Telesimulation is a promising means to improve clinician access to simulation training in infant feeding.

**Supplementary Information:**

The online version contains supplementary material available at 10.1007/s00455-024-10746-7.

## Introduction

Simulation is a training modality widely used in healthcare to support learners to develop competence and confidence by providing opportunities to apply clinical skills in an environment that replicates the clinical setting. Traditionally, simulated learning experiences have been provided in an in-person setting, where learners can interact directly with low-fidelity models (e.g., mannequins), high fidelity models (e.g., Human Patient Simulators), or standardized patients [1]. Unfortunately, in-person simulation limits accessibility for practitioners who are unable to attend these opportunities due to travel requirements. The COVID-19 pandemic also significantly limited capacity to provide in-person training [2]. To counter some of the challenges experienced with accessibility to in-person simulation, “telesimulation” has emerged.

Telesimulation provides simulated learning experiences via telepractice, with learners accessing the simulation experience remotely from different locations [3]. The use of telesimulation has been explored in medical and nursing settings [4], and with allied health students [5], and learners generally report high levels of satisfaction and self-efficacy with this training modality [6]. However, to date, only relatively few studies have compared the outcomes of telesimulation to traditional in-person simulation, with most studies conducted within the medical profession. Results suggest that similar learning outcomes can be achieved between in-person simulation and telesimulation when the learner completes discrete tasks, such as chest drain insertion [7, 8]. McCoy et al. (2017) completed a prospective crossover randomized controlled trial using two immersive scenarios with critically ill patients delivered in both modalities, and found that medical students achieved similar outcomes and satisfaction levels regardless of learning modality. However, the telesimulation condition in that study involved the tele-participants observing the in-person practitioners and participating in a remote debriefing session, rather than actively practicing the clinical skills themselves [9]. The learner experience of in-person simulation versus telesimulation has also been found to be comparable. Nomura et al. (2023) explored the emotional reactions experienced by medical students in telesimulation and found that these were no different to those experienced during in-person simulation [10]. In contrast, a study examining self-reported improvements in ‘comfort’ for managing critical care scenarios found that medical students in the telesimulation environment reported smaller improvements, however, also reported that the learners were highly satisfied regardless [11].

Within the field of speech pathology, training in pediatric feeding management has historically been reported as lacking, with many clinicians describing low confidence and lack of readiness to practice in this area [12, 13]. Accessible remote learning opportunities do exist, but many courses offer only lectures or case-based learning, rather than the opportunity to explicitly practice the application of clinical skills. Research has demonstrated that interactive learning opportunities such as simulation offer the greatest benefit for improving clinical practice and changing patient outcomes [14], over and above didactic training opportunities [15]. Previous research in simulation for pediatric feeding training has been conducted with speech pathology students using in-person simulation, with students identifying increased confidence and knowledge [16], and clinical reasoning skills [17]. With the theory that implementing telesimulation may improve access to practical training opportunities in pediatric feeding, the current team have explored the feasibility of using this teaching modality for this topic, with clinicians indicating high satisfaction levels following exposure to telesimulation [18]. Another pilot study has explored the use of telesimulation for teaching lactation skills to medical students, and improvements in knowledge with high satisfaction were observed [4]. However, despite the demonstration of feasibility for telesimulation in pediatric feeding, there have been no studies specifically comparing the learning outcomes in telesimulation to those experienced in an in-person setting.

The primary aim of this study, therefore, was to compare the effectiveness of in-person simulation to telesimulation for infant feeding training with regards to clinical reasoning, self-perceived confidence, self-perceived anxiety, and satisfaction. A secondary aim was to compare the learning outcomes of self-identified novice practitioners to self-identified experienced practitioners. It was hypothesized that telesimulation would result in similar learning outcomes with regard to clinical reasoning, confidence and anxiety levels when compared to in-person simulation, and that participants would report similar satisfaction levels for both modalities. For the secondary aim, it was hypothesized that novice learners would report larger benefits than experienced participants in clinical reasoning skills and self-perceived confidence and anxiety regardless of randomization allocation.

## Methods

### Trial Design

This study used a pragmatic randomized controlled trial design. Ethical clearance was provided from the Children’s Health Queensland Hospital and Health Service Human Research Ethics Committee (HREC/21/QCHQ/80217) and The University of Queensland (2021/HE002620).

### Participants

Participants were recruited via an expression of interest (EOI) that was disseminated via email across existing professional networks. Written consent was obtained through the EOI. To be eligible to participate in the study, participants had to (a) be a qualified speech pathologist (i.e., not a student); (b) have potential to, or already be seeing children with pediatric feeding disorder; (c) work in Queensland, Australia; (d) have the capacity to attend the in-person simulation at the Queensland Children’s Hospital for the purposes of randomization; and (e) meet COVID-19 vaccination requirements for onsite visits at the research facility. During the EOI process, participants self-identified as either experienced (> 6 months experience with infant feeding) or novice (< 6 months experience with infant feeding).

### Interventions

Participants were randomized to participate in either an in-person simulation or a telesimulation. The same simulated scenario was used for both modalities, with adaptations made for the telesimulation condition. The same facilitators were used for all sessions. The feasibility and acceptability of telesimulation using the same scenario was demonstrated in a previous study, using an iterative process of user testing and adaptation [18].

The simulation involved a 3-week old infant with laryngomalacia, and the session included five components: a pre-brief, didactic teaching, part-task activities, pause-discuss scenario, and debrief. All participants received the same pre-learning materials ahead of the simulation to support their preparation. Each group contained a maximum of six participants and included a mixture of self-identified novice and experienced participants. Sessions were targeted at the novice level learners and were delivered using the same content during each session. Further detail regarding simulation content can be found in a previous paper [18]. The simulations delivered in both conditions aligned with the Healthcare Simulation Standards of Best Practice [19].

For the in-person condition, participants attended the session on site at the Queensland Children’s Hospital, completing the simulation as a group over 4 h in a single room. Due to COVID-19 restrictions, social distancing was applied, and face masks were worn during the sessions. Due to their in-person presence, participants were able to manually handle the mannequins and equipment (e.g., bottle nipples) during the part-task activities.

For the telesimulation condition, participants were sent a Zoom^®^ link ahead of their scheduled 4-hour session. It was requested that they link in individually from a quiet room using a computer with audiovisual capacity. They were asked to have a doll or plush toy available at the time of the simulation and used this to practice oral reflex examination and positioning during the part-task activities (as a substitute for the mannequin used in the in-person simulation). Exploration of different bottle nipples was done visually using shared images over Zoom^®^.

### Outcomes

Each participant was assigned a unique identifier for use throughout the study, to allow for outcomes collected at different points to be compared. Outcomes were collected across three different time points: pre-simulation, immediately post-simulation, and at 4-weeks post-simulation. The primary outcome was clinical reasoning, with secondary outcomes as self-perceived confidence and anxiety, and satisfaction.

#### Demographic Questionnaire

At the commencement of the study, a demographic questionnaire was used to collect information regarding each participant’s workplace context, and their previous experiences and/or training in infant feeding. The questionnaire included 16 questions (13 multiple choice, and 3 open-ended), and was adapted from a previous study [20].

#### Clinical Reasoning

A clinical vignette was used to assess change in clinical reasoning skills pre- and post-simulation. A written case scenario was presented to the participants, and they were asked to write everything they would do with the patient at that time, including any clinical tasks, future planning, considerations for family-centred care, and onward referrals. The same vignette was presented at the pre- and immediately post- timepoints, but a different vignette was presented at the 4-week post timepoint. The 4-week post vignette was matched to the original vignette according to approximate age of the case, and number of significant features with regards to family situation, relevant co-morbidities, and feeding context cues. The purpose of the new vignette was to assess whether participants could transfer their learnt skills to a new case. The clarity and authenticity of the clinical vignettes was checked by three expert clinicians at the Queensland Children’s Hospital, and ideal answers were developed.

A marking rubric was designed with precedence from another study regarding pediatric feeding simulation [17]. The clinical vignettes were marked against five criteria: clinical tasks (i.e., What do I need to do?), clinical reasoning (i.e., Why do I need to do the tasks I selected?), planning (i.e., Did I consider the bigger picture and what might happen next?), family-centred care (i.e., Did I involve the family/patient in my decision-making?), and interprofessional care (i.e., What is my job? What did I need to ask someone else to do? Did I ask the right person?). Each criteria included four levels of attainment: minimal, developing, competent, and mastery. Clear instructions for meeting each level were developed, leading to a total possible score of 20. A copy of the clinical vignettes and the marking rubric is available as supplementary material (Appendix A). Each vignette was then piloted with three novice clinicians at the Queensland Children’s Hospital to refine the marking criteria. Marking of the vignettes was completed independently by two members of the research team who were blinded to allocation and timepoint where possible (i.e., it was not possible to blind markers to the third timepoint, which used a different vignette). A third party reviewed any disagreements in scoring.

#### Self-perceived Confidence and Anxiety Questionnaire

Participants were asked to rate their confidence and anxiety with regards to infant feeding care at all three timepoints, using a questionnaire adapted from a previous study [21]. The questionnaire included two visual analogue scales, where participants were asked to rate their overall confidence and anxiety from 0 to 100, where 0 = not confident/not anxious at all, and 100 = very confident/extremely anxious. The questionnaire also included a series of statements where participants were asked to rate their agreement along a 5-point Likert scale (e.g., *“I am confident in my ability to identify infants who need care for feeding disorders and/or dysphagia”*). Participants’ perceptions of the impact of simulation on their confidence and anxiety were captured in two additional questions in the questionnaires completed post-simulation. An additional question at these timepoints also explored participants’ perceptions of the impact of specific simulation components.

#### Satisfaction

A satisfaction questionnaire was completed immediately post-simulation and was developed from a previous study [21]. This questionnaire included six statements, and participants were asked to indicate their agreement with each statement along a 5-point Likert scale (e.g., *“Involvement in the simulated learning experience was a worthwhile experience for me”*). Participants were also asked to rate their satisfaction with each simulation component.

### Sample Size

The extent of reasonable change was estimated using the scoring of the primary outcome measure of clinical reasoning from a previous paper, where a change score of 2.0 ± 1.5 points for the overall rating on the clinical vignettes was considered clinically important [17]. Power calculations indicated that a sample size of 16 participants per group was required in order to detect a statistically significant change of at least 2.0 points in clinical reasoning scores (power = 0.90, α = 0.01; d = 0.8; nQuery version 9.3.1).

### Randomization

Block randomization (2 novice to 1 experienced) was used to allocate participants to their respective simulation modality to achieve a balance of experience levels across modalities. This ratio was determined according to the number of experienced vs. novice participants who completed an expression of interest. Including participants with different experience levels in the simulation was identified as valuable in our feasibility study [18]. A computer-generated random number sequence was used to generate the allocations. Allocation was completed using concealed envelopes by a neutral party external to the study.

### Blinding

Due to the nature of the program provided, it was not possible to blind the researchers providing the simulations, nor was it possible to blind the participants to their allocated modality.

### Fidelity

Fidelity checks were completed at the end of each simulation session that included descriptive details regarding the number of participants who attended, the ratio of novice to experienced participants, and the structure of the session, to ensure that the simulations provided were the same across groups. Details regarding structure of the session included whether the essential elements of simulation were provided, comprising a pre-brief, didactic, part-task activities regarding respiratory distress, vital signs identification, and infant feeding management, and the simulation itself. Information captured regarding the simulation itself included the use of visual elements (backgrounds, costumes, and cognitive aids), whether modelling from an experienced clinician was provided, and whether the two facilitator roles were kept as distinct and separate during the simulation itself. Finally, the length of the debrief provided at the end of each session was recorded.

### Statistical Analyses

The Statistical Package for the Social Sciences (SPSS, version 27) was used for data analysis. Data were presented predominantly using descriptive statistics. In seven cases, the final 4-week post datapoint was missing. In order to prevent bias via the exclusion of missing data, data was imputed using the missing participants’ previous scores [22]. Where data were not normally distributed, a transformation using the square root of the data was completed, and normality confirmed [23]. This was conducted for the variable of overall confidence. Groups were compared at baseline using a chi-square test to examine differences in proportions, and an independent t-test where data were continuous and normally distributed.

Inferential statistics were used to explore the primary outcome measure of clinical reasoning, as measured by the overall scores on the clinical vignettes. Differences between groups and across timepoints were assessed using a two-way mixed analysis of variance (ANOVA) following confirmation of normal distribution using the Kolmogorov-Smirnov test. This testing was repeated for the variables of overall confidence and anxiety ratings, using the transformed data for confidence ratings to ensure normal distribution. No assumptions were violated and there were no significant outliers in the data. Ordinal data in the individual confidence statement ratings were compared across timepoints using a Friedman test, and a Mann-Whitney U test to examine specific differences according to randomization and experience levels. Satisfaction data between groups were compared using a t-test, following confirmation of normal distribution. To control for the testing of multiple hypotheses, a conservative *p*-value of 0.01 was applied.

## Results

### Participant Demographics

**Fig. 1:**
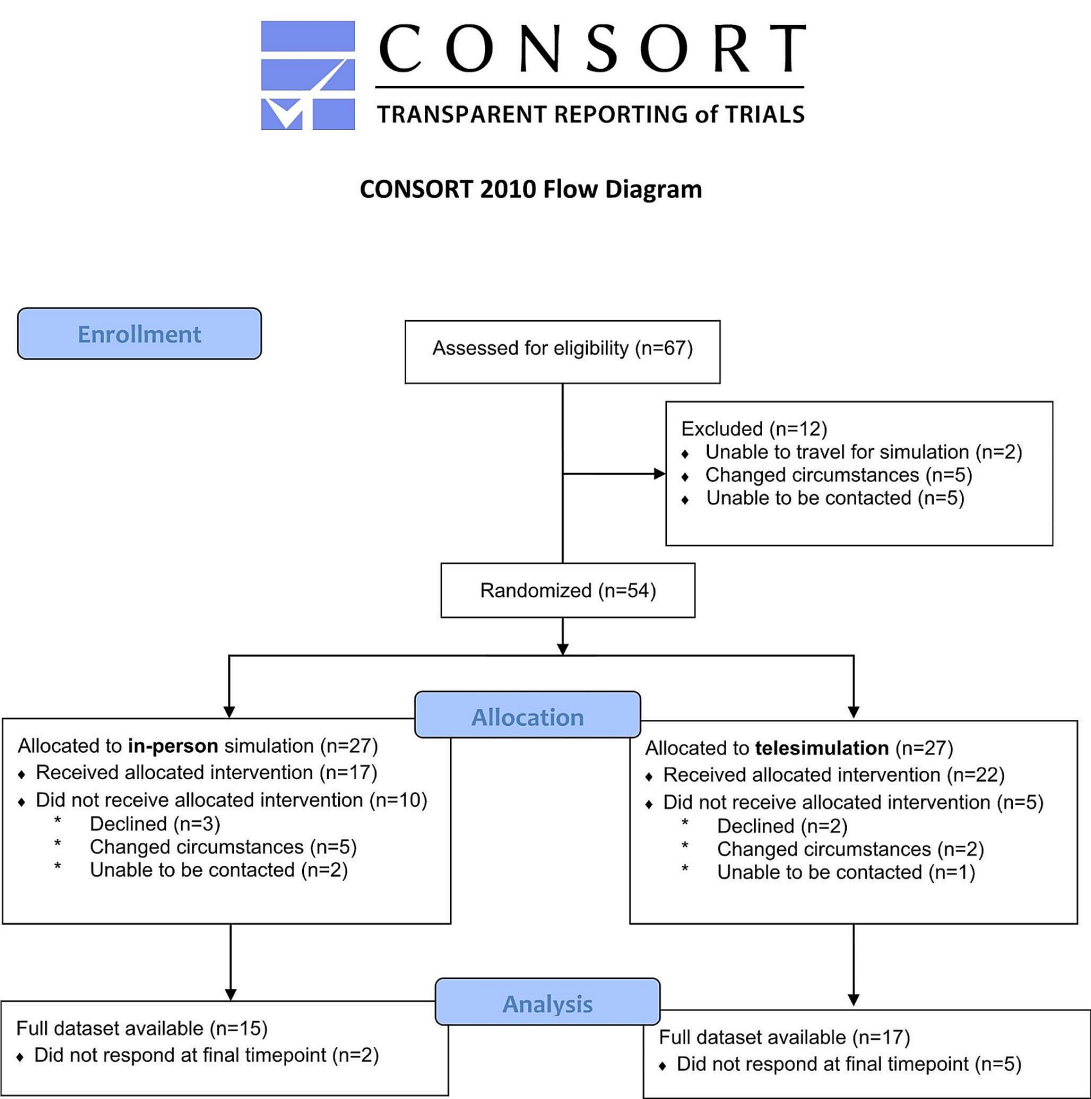
CONSORT 2010 flow diagram

Overall, 67 speech pathologists expressed interest in participating in the study, and of these, 54 met eligibility criteria and were randomized (*n* = 27 in-person; *n* = 27 telepractice) (Fig. 1). A COVID-19 outbreak delayed the provision of the simulation sessions after randomization by four months. Of those who were randomized, 39 progressed to participation in their allocated simulation modality (*n* = 17 in-person; *n* = 22 telepractice). Reasons for non-participation included declining to participate without a specific reason (*n* = 5), changed work circumstances (*n* = 7), and being unable to be contacted (*n* = 3). There were seven participants who did not complete the final timepoint measure after the simulation, and their data was analysed using an intention-to-treat analysis. A greater proportion of those that failed to complete the final data timepoint were novice practitioners (*n* = 6, 86%).

Of the participants where data was analysed, a total of 16 (41%) identified as experienced, and 23 (59%) identified as novices. Participants described working across several settings, including a hospital (*n* = 9, 23%), community health service (*n* = 8, 21%), private practice (*n* = 12, 31%), disability service (*n* = 7, 18%), school (*n* = 1, 3%), mental health service (*n* = 1, 3%), and university (*n* = 1, 3%). Even though some participants had provided infant feeding services in the past, most participants reported that they never or rarely provided infant feeding services at the time of the simulation (*n* = 26, 67%).

### Fidelity Checks

There were 9 simulation sessions conducted in total (*n* = 5 telesimulation; *n* = 4 in-person), and all were 4 h in length with one 15-minute break. The total number of participants for each modality ranged from 3 to 6 participants for telesimulation, and 2 to 6 participants for in-person simulation. In one of the in-person simulations, there were no ‘experienced’ participants in attendance (as they were unable to attend due to illness), so the facilitator provided modelling and scripting during the session for the participants to ensure this component of teaching was met. All other simulation components were provided consistently across all scenarios i.e., pre-brief, didactic, part-tasks, simulation backgrounds, costumes, and visual aids. Debrief sessions ranged from 10 to 20 min in length (mean 13.8 min).

### Comparison of In-person and Telesimulation Groups at Baseline

Table 1 provides baseline information regarding the two groups of participants. There were no significant differences between the groups in the proportion of novice to experienced participants, in infant feeding experience, or across the measures used in the study. For those that had accessed simulation previously, the majority had experienced this as an undergraduate student (*n* = 16, 41%), as compared to those who had experienced simulation in the workplace (*n* = 8, 21%).

**Table 1:** Differences between groups pre-simulation

Variable	In-person simulation (*n* = 17)	Telesimulation (*n* = 22)	*p*-value
Experienced, n (%)	7 (41%)	9 (41%)	*p* = 1.000*
Novice, n (%)	10 (59%)	13 (59%)	
Currently work with infants < 6 months, n (%)	4 (24%)	10 (46%)	*p* = 0.281*
Previously done infant feeding professional development, n (%)	10 (59%)	16 (73%)	*p* = 0.568*
Parenting or childcare experience, n (%)	6 (35%)	11 (50%)	*p* = 0.553*
Accessed simulation as a teaching modality previously, n (%)	10 (59%)	14 (64%)	*p* = 1.000*
Overall self-rated confidence/100 (mean, SD)	26.1 (± 27.3)	28.2 (± 29.6)	*p* = 0.821^
Overall self-rated anxiety/100 (mean, SD)	59.0 (± 27.6)	54.4 (± 33.2)	*p* = 0.648^
Overall score on clinical vignette measure/20 (mean, SD)	10.9 (± 3.1)	12.8 (± 3.3)	*p* = 0.073^

*Result of chi-square test; ^Result of independent samples t-test

### Primary Outcome

#### Clinical Reasoning

Examination of the main effect for time within groups for clinical reasoning indicated a significant improvement across timepoints overall for all participants (F_2, 74_=10.386, *p* < 0.001, partial ƞ^2^=0.219) (see Table 2). Post-hoc testing revealed that this change was significant between the pre- and 4-weeks post-timepoints (*Mean difference =* 2.00; CI -3.03 to − 0.90; *p* < 0.001), but not the pre- and post (*Mean difference =* 1.20; CI -2.41 to − 0.02; *p* = 0.046) or post to 4-weeks post timepoints (*Mean difference =* 0.80; CI -1.76 to 0.25; *p* = 0.206).

**Table 2:** Within- and between-groups comparisons for clinical reasoning, self-reported confidence and anxiety

Outcome	Pre- mean (± SD)	Post- mean (± SD)	4-weeks post mean (± SD)	Interaction between time and randomization		Main effect of time (within groups)		Main effect of randomisation (between groups)	
				*p*-value	Partial ƞ^2^	*p*-value	Partial ƞ^2^	*p*-value	Partial ƞ^2^
**Clinical Reasoning/20**				0.718	0.009	< 0.001	0.219	0.033	0.12
All participants	11.95 (± 3.28)	13.15 (± 3.44)	13.95 (± 3.26)						
In-person simulation	10.88 (± 3.10)	12.18 (± 3.66)	12.59 (± 3.26)						
Telesimulation	12.77 (± 3.27)	13.91 (± 3.13)	15.00 (± 2.91)						
**Overall self-reported confidence/100**				0.705	0.005	< 0.001	0.594	0.334	0.025
All participants	27.31 (± 28.26)	55.85 (± 20.98)	57.90 (± 19.37)						
In-person simulation	26.12 (± 27.32)	50.47 (± 20.06)	54.00 (± 18.88)						
Telesimulation	28.23 (± 29.57)	60.00 (± 21.18)	60.91 (± 19.64)						
**Overall self-reported anxiety/100**				0.430	0.023	< 0.001	0.336	0.966	0.000
All participants	56.41 (± 30.59)	37.23 (± 22.46)	38.46 (± 20.67)						
In-person simulation	59.00 (± 27.64)	34.53 (± 21.94)	38.06 (± 16.36)						
Telesimulation	54.41 (± 33.19)	39.32 (± 23.14)	38.77 (± 23.85)						

Between groups, the telesimulation group demonstrated a greater extent of change overall in clinical reasoning than the in-person group (*Mean difference telesim =* 2.23; *Mean difference in-person =* 1.71; F_1,37_=4.935; *p* = 0.033; partial ƞ^2^=0.12) (Table 2) although this difference was not considered statistically significant. For the telesimulation group, this change was not significant pre- to post- (*Mean difference* = 1.14, *SD* = 2.90; t(21) = 1.84, *p* = 0.08) or post-to 4-weeks post (*Mean difference* = 1.09, *SD* = 2.62; t(21) = 1.96, *p* = 0.06). The change was also not significant for the in-person group pre- to post- (*Mean difference* = 1.30, *SD* = 3.04; t(16) = 1.78, *p* = 0.10) or post- to 4-weeks post (*Mean difference* = 0.41, *SD* = 2.29; t(16) = 0.74, *p* = 0.47).

### Secondary Outcomes

#### Self-perceived Confidence and Anxiety

The assumption of sphericity was violated for the two-way interaction regarding overall self-reported confidence (χ^2^(2) = 38.71; *p* < 0.001) and subsequently, the Greenhouse-Geisser correction was used to interpret the results. Overall, for the main effect of time within groups, self-perceived confidence increased across the three timepoints, and this change was significant (*F*_1.21,44.61_=54.07; *p* < 0.001, partial ƞ^2^=0.594; ε = 0.603). Sub-analyses of the change across timepoints revealed that the change in self-perceived confidence was significant pre- to post- (*Mean difference =* 28.54; CI 19.12 to 37.00; *p* < 0.001), and pre- to 4-weeks post (*Mean difference =* 30.28; CI 21.65 to 38.92; *p* < 0.001), but not post- to 4-weeks post (*Mean difference =* 2.05; CI -1.28 to 5.72; *p* = 0.198). There was no significant difference in improvements in overall self-perceived confidence measures between the in-person simulation and telesimulation groups (*Mean difference telesim =* 32.68; *Mean difference in-person =* 27.88; *F*_1,37_=0.958; *p* = 0.334; partial ƞ^2^=0.025). Changes across all individual confidence statements were significant (Table 3), regardless of simulation modality.

**Table 3:** Confidence statements

Statement		Pre- median (± IQR)	Post- median (± IQR)	4-weeks post median (± IQR)	Across groups	Overall change
I am confident in my ability to identify infants who need care for feeding disorders and/ or dysphagia	In person simulation	4(± 1)	4(± 1)	4(± 1)	χ^2^ = 13.54 *p* < 0.01	χ^2^ = 28.79 *p* < 0.01
	Telesimulation	3.5(± 2)	4(± 0.75)	4(± 0)	χ^2^ = 16.15 *p* < 0.01	
I am confident in my ability to assess infants with feeding disorders and/ or dysphagia	In person simulation	2(± 1)	4(± 1)	4(± 1)	χ^2^ = 29.43 *p* < 0.01	χ^2^ = 52.54 *p* < 0.01
	Telesimulation	2.5(± 1)	4(± 1)	4(± 1)	χ^2^ = 24.04 *p* < 0.01	
I am confident in my ability to handle an infant	In person simulation	4(± 3)	4(± 2)	4(± 2)	χ^2^ = 15.31 *p* < 0.01	χ^2^ = 19.59 *p* < 0.01
	Telesimulation	4(± 2)	4(± 2)	4(± 1)	χ^2^ = 7.29 *p* < 0.05	
I am confident in my ability to complete an infant oral reflex examination	In person simulation	2(± 2)	4(± 0)	4(± 0)	χ^2^ = 31.76 *p* < 0.01	χ^2^ = 56.17 *p* < 0.01
	Telesimulation	3(± 2)	4(± 0)	4(± 0.75)	χ^2^ = 24.67 *p* < 0.01	
I am confident in my ability to identify different bottles and nipples	In person simulation	2(± 1)	4(± 1)	4(± 1)	χ^2^ = 15.95 *p* < 0.01	χ^2^ = 35.69 *p* < 0.01
	Telesimulation	3(± 2)	4(± 0.75)	4(± 1)	χ^2^ = 20.65 *p* < 0.01	
I am confident in my ability to identify signs and symptoms of respiratory distress in an infant	In person simulation	3(± 2)	4(± 1)	4(± 0)	χ^2^ = 19.46 *p* < 0.01	χ^2^ = 35.65 *p* < 0.01
	Telesimulation	4(± 1)	4(± 0)	4(± 0.75)	χ^2^ = 14.04 *p* < 0.01	
I am confident in my ability to make decisions around oral feeding in an infant with respiratory distress	In person simulation	2(± 1)	4(± 1)	4(± 1)	χ^2^ = 33.25 *p* < 0.01	χ^2^ = 55.81 *p* < 0.01
	Telesimulation	2.5(± 2)	3.5(± 1)	4(± 1)	χ^2^ = 23.11 *p* < 0.01	

Within groups, self-perceived anxiety decreased significantly across the three timepoints regardless of allocated modality (*F*_2,74_=18.719; *p* < 0.001, partial ƞ^2^=0.336). Post-hoc analyses revealed that the reduction in anxiety was significant between the pre- to post- timepoints (*Mean difference=*-19.18; CI -30.04 to -9.53; *p* < 0.001), and the pre- to 4-weeks post timepoints (*Mean difference=*-17.95; CI -27.66 to 8.92; *p* < 0.001), but not between the post- and 4-weeks post timepoints (*Mean difference =* 1.23; CI -5.69 to 8.67; *p* = 1.000). There was no significant difference in improvements in overall self-perceived anxiety between the in-person simulation and telesimulation groups (*Mean difference telesim=*-15.64; *Mean difference in-person=-*20.94; CI -13.87 to 14.48; *F*_1,37_=0.002; *p* = 0.966; partial ƞ^2^=0.000).

#### Satisfaction

Overall, participants reported high levels of satisfaction across both simulation modalities, with a median score of 4 or 5 out of a total of 5 reported across all items. There were no significant differences between the groups across individual items, suggesting similar satisfaction levels between the two groups. No significant differences were observed between the telesimulation and in-person groups regarding the perceived effectiveness of different teaching modalities employed in the simulation (Table 4).

**Table 4:** Satisfaction measures between groups

Statement	Median (± IQR)			
*Satisfaction questionnaire*: *Please provide an answer between 1 and 5*,* where 1 = strongly disagree*,* 3 = neutral and 5 = strongly agree.*	Tele (*n* = 22)	In-person (*n* = 17)	*p*-value	*r*
Involvement in the simulated learning experience was a worthwhile experience for me	5(± 1)	5(± 1)	0.652	0.07
I will recommend simulation to my colleagues	5(± 1)	5(± 0)	0.321	0.16
My participation in the simulated learning experience has enhanced my professional satisfaction	5(± 1)	5(± 1)	0.409	0.13
I believe that the quality of the care I provide my patients has improved because of the simulated learning experience	4(± 1)	4(± 0)	0.185	0.21
The goals and objectives I had upon becoming involved in this learning experience have been met	5(± 0.75)	4(± 1)	0.041	0.33
The simulated learning experience has reduced variations in care	4(± 1)	4(± 1)	0.098	0.26
*How do you think specific components of simulation supported your learning? (where 1 = of no help at all; 3 = of some help; and 5 = helped a huge amount)*				
Didactic presentation	5(± 0.75)	5(± 1)	0.382	-0.14
Part-task learning	5(± 0.75)	5(± 1)	0.871	-0.03
Simulation	5(± 0)	5(± 0)	0.954	-0.01
Debrief	5(± 1)	5(± 1)	0.367	-0.14

### Differences between Experienced and Novice Level Participants

The differences in outcomes between novice and experienced participants were compared. An improvement in the overall score for clinical reasoning was observed across the three timepoints, with the experienced participants demonstrating greater change (*Mean difference experienced* = 2.50; *Mean difference novice* = 1.65; *F*_1,37_=4.51; *p* = 0.040; partial ƞ^2^=0.11), although this result was not considered statistically significant. When self-perceived confidence and anxiety were examined, novice level participants reported significantly greater changes across both confidence (*Mean difference experienced =* 18.94; *Mean difference novice* = 38.70; *F*_1, 37_=27.86; *p* < 0.001; partial ƞ^2^=0.43) and anxiety (*Mean difference experienced*=-9.75; *Mean difference novice*=-23.65; F_1,37_=17.30; *p* < 0.001; partial ƞ^2^=0.32) than experienced participants. There were no significant differences between novice and experienced participants for any of the satisfaction statements.

**Table 5:** Differences in outcomes between groups for novice level and experienced participants

Outcome	Pre- mean (± SD)	Post- mean (± SD)	4-weeks post mean (± SD)	*p*-value	Partial eta squared
**Clinical Reasoning (overall)/20**					
Novice (*n* = 23)	11.39 (± 3.12)	12.22 (± 3.20)	13.04 (± 2.87)	0.040	0.109
Experienced (*n* = 16)	12.75 (± 3.44)	14.50 (± 3.41)	15.25 (± 3.44)		
**Overall self-reported confidence/100**					
Novice (*n* = 23)	11.13 (± 14.5)	47.09 (± 14.07)	49.83 (± 15.26)	< 0.001	0.430
Experienced (*n* = 16)	50.56 (± 27.15)	68.44 (± 23.20)	69.50 (± 19.12)		
**Overall self-reported anxiety/100**					
Novice (*n* = 23)	70.22 (± 25.35)	45.13 (± 17.32)	46.57 (± 16.07)	< 0.001	0.319
Experienced (*n* = 16)	36.56 (± 26.74)	25.88 (± 24.58)	26.81 (± 21.40)		

## Discussion

This study presents the results of a pragmatic randomized controlled trial comparing learning outcomes and satisfaction between in-person simulation and telesimulation for infant feeding. Overall, telesimulation was considered equivalent to in-person simulation in effecting improvements to clinical reasoning, improvements to self-perceived confidence, and reductions in self-perceived anxiety. No differences were observed between modalities with regards to individual confidence statements, or with regards to satisfaction with different components of the simulation. General satisfaction was high across both groups, regardless of allocated modality or experience levels. These results are consistent with other research in this area comparing telesimulation to in-person simulation, which has found similar improvements in performance across both teaching modalities in procedural task completion [7, 8], increases in confidence levels [8], and high levels of satisfaction with telesimulation [8, 11, 24].

This research demonstrated that telesimulation is a viable alternative to in-person simulation for pediatric feeding training. Multiple studies have identified low clinician confidence and barriers with access to practical training in pediatric feeding [12, 13], and access to ongoing educational opportunities and practice opportunities with patients are correlated with increased confidence [12, 25]. Given that telesimulation provides increased access to learning opportunities with similar outcomes demonstrated to in-person simulation, it is reasonable to suggest that telesimulation has the potential to fill a significant professional development gap in this field. Additionally, as less practical resourcing and minimal travel is required, telesimulation also has the potential to be cheaper than in-person simulation [26], although comparative costs are yet to be described in the literature. This would be an area for future research.

Knowledge decay is a common issue following professional development. The literature suggests that problem-based learning, where the onus is on practical, skills-based problem solving, is more effective at facilitating knowledge retention than didactic teaching alone [27]. In a clinical setting, knowledge decay may occur when participants are unable to apply or generalize their learnings to a new environment, generally due to limited opportunity. Although the improvements to the primary outcome of clinical reasoning were not significant between each timepoint, overall there was a significant positive effect observed regardless of allocated modality. It could be suggested that the simulation itself promoted some improvements to clinical reasoning that were further consolidated as the participants had the opportunity to apply their learnings in a clinical setting. Whilst it was not possible to control each participants’ local context, the maintenance of clinical reasoning scores and self-perceived confidence and anxiety ratings observed in our study provides a promising outlook for the effectiveness of this teaching modality. Future research could explore a longer follow-up time, opportunities for follow-up simulations for skills maintenance, and/or establishing a community of practice within the participant group to discuss ongoing cases.

The endpoint of any professional development for clinicians sits with impact at the patient level. Our study explored outcomes for participants in a discrete simulation scenario within the bounds of a structured randomized controlled trial and did not extend to exploring outcomes at the patient level. Whilst numerous telesimulation studies explored to date have examined outcomes at the level of the participant in a similar manner to this study [4, 7–11, 24], a recent scoping review identified that no known studies have explored the transfer of skills learnt during telesimulation to clinical practice [6]. Further implementation of this work is required to define best practice in telesimulation and truly measure the impact on patient care.

It was hypothesized that the participants who self-identified as experienced practitioners in this study may have derived less benefit from this training as the case was targeted at the novice level. However, this was not the case and there were no significant differences in satisfaction observed between the groups. The novice level participants self-reported significantly greater changes in confidence and anxiety, which was unsurprising as the experienced participants reported high confidence and/or low anxiety prior to the simulation. It was interesting that the experienced cohort demonstrated greater changes to their clinical reasoning (clinical vignette scores) than the novice group. It is suggested that the experienced group were better able to incorporate new knowledge to meet the expectations of the tool used to assess clinical reasoning. Further exploration of how best to measure changes in clinical reasoning, as well as the perceived value of simulation/telesimulation for experienced participants is warranted.

### Limitations

Overall, 28% (*n* = 15) of randomized participants did not complete their allocated teaching, with twice as many participants lost to follow up in the in-person arm. We surmised that this was contributed to by a COVID-19 surge limiting our capacity to commence data collection by 5 months after randomization, but for those who did not provide a reason for declining or were unable to be contacted, the true reason for dropout is not known. There were also seven participants who did not complete questionnaires at the final datapoint, for whom we used an intention-to-treat analysis. Additionally, although the results revealed equivalent satisfaction scores between tele- and in-person simulation groups, it is possible that satisfaction in both groups may have been influenced by the COVID-19 pandemic and subsequent studies are needed to determine whether satisfaction remains equivalent between groups when the fear and risks of viral spread that existed during the pandemic are not present.

Another limitation of this study is the subjective nature of the measurement tools used. Although as much rigor as possible was applied, the primary outcome measure in this study required subjective interpretation, and it was difficult to determine what constituted a meaningful improvement in scores. This challenge may have impacted the way in which we were able to interpret the primary outcome measure in this study; significant differences may have been observed had the measure been more sensitive to change. In addition, we did not control for any further activities that the learners may have accessed in order to consolidate their learning between the post- and 4-weeks post timepoints. As a result of this, it is difficult to conclude definitively that simulation alone effected a change in clinical reasoning outcomes, but rather that it initiated a learning pathway. This study also relied on self-reported measures of confidence and anxiety, which are known to have limitations [28, 29]. Consideration of these factors should be taken into account when interpreting the results.

## Conclusions

This study compared the learning outcomes and satisfaction between in-person simulation and telesimulation for infant feeding, demonstrating that telesimulation and in-person simulation facilitated similar improvements in clinical reasoning, and had similar outcomes for improvements in self-perceived confidence, and reductions in self-perceived anxiety. Participants reported high satisfaction with simulation regardless of their allocated education modality or experience levels. Telesimulation presents as a promising option for improved accessibility to practical training for pediatric feeding. Further exploration into implementation and the impact on patient care is now required.

## Electronic Supplementary Material

Below is the link to the electronic supplementary material.

Supplementary Material 1

## Data Availability

Raw data for this project are not publicly available in order to preserve participants’ privacy.

## References

[1] Lopreiato JO, Sawyer T. Simulation-based medical education in pediatrics. Acad Pediatr. 2015;15(2):134–42. 10.1016/j.acap.2014.10.010 25748973 10.1016/j.acap.2014.10.010

[2] Diaz MCG, Walsh BM. Telesimulation-based education during COVID-19. Clin Teach. 2021;18(2):121–5. 10.1111/tct.13273 33043589 10.1111/tct.13273 PMC7675436

[3] McCoy CE, Sayegh J, Alrabah R, Yarris LM. Telesimulation: an innovative tool for health professions education. AEM Educ Train. 2017;1(2):132–6. 10.1002/aet2.10015 30051023 10.1002/aet2.10015 PMC6001828

[4] Anderson OS, Weirauch K, Roper R, Phillips J, McCabe C, Chuisano SA, et al. The efficacy of hybrid telesimulation with standardized patients in teaching medical students clinical lactation skills: a pilot study. Breastfeed Med. 2021;16(4):332–7. 10.1089/bfm.2020.0253 33493401 10.1089/bfm.2020.0253

[5] Howells S, Cardell EA, Waite MC, Bialocerkowski A, Tuttle N. A simulation-based learning experience in augmentative and alternative communication using telepractice: speech pathology students’ confidence and perceptions. Adv Simul (Lond). 2019;4(Suppl 1):23. 10.1186/s41077-019-0113-x 31890318 10.1186/s41077-019-0113-x PMC6924137

[6] Heffernan R, Brumpton K, Randles D, Pinidiyapathirage J. Acceptability, technological feasibility and educational value of remotely facilitated simulation based training: a scoping review. Med Educ Online. 2021;26(1):1972506. 10.1080/10872981.2021.1972506 34433385 10.1080/10872981.2021.1972506 PMC8405121

[7] Falcioni AG, Yang HC, de Mattos ESE, Maricic MA, Ruvinsky S, Bailez MM. Comparative effectiveness of telesimulation versus standard simulation for pediatric minimally invasive surgery (MIS) essential skills training. J Pediatr Surg. 2023;58(4):669–74. 10.1016/j.jpedsurg.2022.12.013 36658075 10.1016/j.jpedsurg.2022.12.013 PMC9773740

[8] Jewer J, Parsons MH, Dunne C, Smith A, Dubrowski A. Evaluation of a mobile telesimulation unit to train rural and remote practitioners on high-acuity low-occurrence procedures: pilot randomized controlled trial. J Med Internet Res. 2019;21(8):e14587. 10.2196/14587 31389340 10.2196/14587 PMC6701160

[9] McCoy CE, Sayegh J, Rahman A, Landgorf M, Anderson C, Lotfipour S. Prospective randomized crossover study of telesimulation versus standard simulation for teaching medical students the management of critically ill patients. AEM Educ Train. 2017;1(4):287–92. 10.1002/aet2.10047 30051046 10.1002/aet2.10047 PMC6001816

[10] Nomura O, Sunohara M, Watanabe I, Itoh T. Evaluating emotional outcomes of medical students in pediatric emergency medicine telesimulation. Child (Basel). 2023;10(1). 10.3390/children10010169 10.3390/children10010169 PMC9856926 36670719

[11] Lin E, You AX, Wardi G. Comparison of in-person and telesimulation for critical care training during the COVID-19 pandemic. ATS Sch. 2021;2(4):581–94. 10.34197/ats-scholar.2021-0053OC 35083463 10.34197/ats-scholar.2021-0053OC PMC8787731

[12] Raatz M, Marshall J, Ward EC, Dickinson C, Frederiksen N, Reilly C, et al. Understanding training needs in pediatric feeding for allied health professionals: an Australian perspective. Am J Speech Lang Pathol. 2023;32(2):452–68. 10.1044/2022_ajslp-22-00232 36692930 10.1044/2022_AJSLP-22-00232

[13] Zimmerman E. Pediatric dysphagia: a rise in preterm infants and a need for formal training for speech-language pathologists. Int J Gynecol Obstet Neonatal. 2016;3:15–20. 10.15379/2408-9761.2016.03.01.03

[14] Piot MA, Dechartres A, Attoe C, Jollant F, Lemogne C, Layat Burn C, et al. Simulation in psychiatry for medical doctors: a systematic review and meta-analysis. Med Educ. 2020;54(8):696–708. 10.1111/medu.14166 32242966 10.1111/medu.14166

[15] Bloom BS. Effects of continuing medical education on improving physician clinical care and patient health: a review of systematic reviews. Int J Technol Assess Health Care. 2005;21(3):380–5. 10.1017/s026646230505049x 16110718 10.1017/s026646230505049x

[16] Clinard ES. Increasing student confidence with medically complex infants through simulation: a mixed methods investigation. Am J Speech Lang Pathol. 2022;31(2):942–58. 10.1044/2021_ajslp-21-00234 35226538 10.1044/2021_AJSLP-21-00234

[17] Miles A, Friary P, Jackson B, Sekula J, Braakhuis A. Simulation-based dysphagia training: teaching interprofessional clinical reasoning in a hospital environment. Dysphagia. 2016;31(3):407–15. 10.1007/s00455-016-9691-0 26803776 10.1007/s00455-016-9691-0

[18] Marshall J, Raatz M, Ward EC, Penman A, Beak K, Moore M et al. Development and pilot testing of telesimulation for pediatric feeding: a feasibility study. Dysphagia. 2023:1–15 10.1007/s00455-023-10556-3 10.1007/s00455-023-10556-3 PMC9872075 36692653

[19] Watts PI, McDermott DS, Alinier G, Charnetski M, Ludlow J, Horsley E, et al. Healthcare simulation standards of best PracticeTM simulation design. Clin Simul Nurs. 2021;58:14–21. 10.1016/j.ecns.2021.08.009

[20] Ward EC, Hill AE, Nund RL, Rumbach AF, Walker-Smith K, Wright SE et al. Developing clinical skills in paediatric dysphagia management using human patient simulation (HPS). International Journal of Speech-Language Pathology: Selected papers from the 2014 Speech Pathology Australia National Conference. 2015;17(3):230–40 10.3109/17549507.2015.1025846 10.3109/17549507.2015.1025846 25833074

[21] Furlan AD, Zhao J, Voth J, Hassan S, Dubin R, Stinson JN, et al. Evaluation of an innovative tele-education intervention in chronic pain management for primary care clinicians practicing in underserved areas. J Telemed Telecare. 2019;25(8):484–92. 10.1177/1357633x18782090 29991316 10.1177/1357633X18782090

[22] Carpenter J, Kenward MG, editors. Missing data in randomised controlled trials: a practical guide. 2007.

[23] Bland JM, Altman DG. Statistics notes: transforming data. BMJ. 1996;312(7033):770. 10.1136/bmj.312.7033.770 8605469 10.1136/bmj.312.7033.770 PMC2350481

[24] Gerstenberger JP, Hayes L, Chow CJ, Raaum S. Medical student experiential learning in telesimulation. J Med Educ Curric Dev. 2023;10:23821205231216067. 10.1177/23821205231216067 38025030 10.1177/23821205231216067 PMC10664437

[25] O’Donoghue CR, Dean-Claytor A. Training and self-reported confidence for dysphagia management among speech-language pathologists in the schools. Lang Speech Hear Serv Sch. 2008;39(2):192–8. 10.1044/0161-1461(2008/019) 18420522 10.1044/0161-1461(2008/019)

[26] Ward EC, Caird E, Khanal S, Kularatna S, Byrnes J, Penman A, et al. A cost analysis of a 5-day simulation-based learning program for speech-language pathology student training. Int J Speech Lang Pathol. 2022:1–9. 10.1080/17549507.2022.2115138 10.1080/17549507.2022.2115138 36062806

[27] Trullàs JC, Blay C, Sarri E, Pujol R. Effectiveness of problem-based learning methodology in undergraduate medical education: a scoping review. BMC Med Educ. 2022;22(1):104. 10.1186/s12909-022-03154-8 35177063 10.1186/s12909-022-03154-8 PMC8851721

[28] Dunning D, Heath C, Suls JM. Flawed self-assessment: implications for health, education, and the workplace. Psychol Sci Public Interest. 2004;5(3):69–106. 10.1111/j.1529-1006.2004.00018.x 26158995 10.1111/j.1529-1006.2004.00018.x

[29] Ehrlinger J, Johnson K, Banner M, Dunning D, Kruger J. Why the unskilled are unaware: further explorations of (absent) self-insight among the incompetent. Organ Behav Hum Decis Process. 2008;105(1):98–121. 10.1016/j.obhdp.2007.05.002 19568317 10.1016/j.obhdp.2007.05.002 PMC2702783

