# Supplementary material for: Telesimulation for Training in Infant Feeding: A Randomized Controlled Trial
Source: Dysphagia. 2024 Aug 12;40(2):443–53. doi: 10.1007/s00455-024-10746-7 (PMC11893704; doi:10.1007/s00455-024-10746-7)
Supplement: Supplementary file 1 — Supplementary Material 1 [file 455_2024_10746_MOESM1_ESM.docx]

**Supplementary Appendix A: Clinical vignettes and marking rubric**

**Clinical vignettes**

**Instructions:**

Describe in one paragraph everything you would do with this patient today and what factors you would consider in your management of this patient. Consider which clinical tasks you would need to do and why, your future planning, how you would integrate family-centred care into your session, and any onward referrals you might need to make.

***Vignette 1 (pre- and immediately post)***

You have an appointment to see a 3-month-old infant, Max. Max, the first child of Lucy and Mark, has recently been diagnosed with laryngomalacia. There have been ongoing concerns regarding feeding, which has been a very stressful experience, particularly for Lucy. Max is currently fully orally fed via bottle. Lucy and Mark typically provide the feed in a cradle hold position, using a Tommee Tippee teat (#2). Lucy reports Max demonstrates significant stridor with feeds and will constantly detach from the teat crying. They have trialled lots of different bottles and teats. Max is well engaged with an ENT who is keen for your review. Max has also recently commenced on reflux medication.

***Vignette 2 (4-weeks post)***

You have an appointment to see a 4-month old infant (corrected age), Gracie, who was born prematurely at 28 weeks and who has Chronic Neonatal Lung Disease (CNLD), for which she has required oxygen supplementation. Gracie is the first child to Ava and George, who have been closely monitoring her oral intake and her development. Over the past few weeks, Gracie has started demonstrating anterior spill, coughing, and choking with feeds, and is beginning to refuse the bottle. She is currently using a Pigeon medium flow standard neck teat due to her age and is fed in a cradle hold position. The medical team are concerned about Gracie’s growth and safety of oral feeds and are querying nasogastric tube re-insertion.

**Rubric***

*Definitions:*

1. *Clinical tasks:* What do I need to do?
2. *Clinical reasoning:* Why do I need to do the tasks I selected?
3. *Planning*: Did I consider the bigger picture and what might happen next?
4. *Patient and family centred care:* Did I involve the family/ patient in my decision-making?
5. *Interprofessional collaboration:* What is my job? What do I need to ask someone else to do? Did I ask the right person for help with this case?

| **Criteria** | **Minimal (1)** | **Developing (2)** | **Competent (3)** | **Mastery (4)** |
| --- | --- | --- | --- | --- |
| Clinical tasks | Does not identify any of the reasonable clinical tasks | Identifies some of the reasonable clinical tasks | Identified most of the reasonable clinical tasks | All reasonable clinical tasks are identified |
| Clinical reasoning | No evidence of aspects of clinical reasoning or EBP applied | Evidence of some aspects of clinical reasoning/ EBP applied | Evidence of most aspects of clinical reasoning/ EBP applied | Evidence of all known aspects of clinical reasoning/ EBP applied |
| Planning | No consideration of overall situation and next steps | Some consideration of overall situation and next steps | Good consideration of overall situation and next steps | Excellent consideration of overall situation and next steps |
| Patient and family centred care | No evidence of information sharing or involving family in decision-making | Some evidence of information sharing or involving family in decision-making | Good evidence of information sharing or involving family in decision-making | Substantial evidence of information sharing or involving family in decision-making |
| Interprofessional collaboration | Does not describe own role to family. Describes clinical tasks that are outside of scope of practice. Does not refer or seek information from other professionals where appropriate | Some reference to own role. Some demonstration of understanding of own scope of practice  Refers or seeks information from other professionals some of the time where appropriate | Good reference to own role. Good demonstration of understanding of own scope of practice  Refers or seeks information from other professionals most of the time where appropriate | Thorough reference to own role and role of others. Demonstrated understanding of scope of practice appropriate point of referral.  Refers or seeks information from other professionals all of the time where appropriate. |

*Developed with reference to Miles, A., Friary, P., Jackson, B., Sekula, J. & Braakhuis, A. (2016). Simulation-based dysphagia training: Teaching interprofessional clinical reasoning in a hospital environment. *Dysphagia, 31,* 407-415.
